# Treatment of HCV Patients Before and After Renal Transplantation

**DOI:** 10.5812/kowsar.1735143X.712

**Published:** 2011-11-30

**Authors:** Ling-yao Du, Hong Tang

**Affiliations:** 1 Center of Infectious Diseases, West China Hospital, Sichuan University, Chengdu, Sichuan, China; 2 Division of Infectious Diseases, State Key Laboratory of Biotherapy, Sichuan University, Chengdu, China

**Keywords:** Kidney Transplantation, Hepatitis C, Antiviral Agents, Immunosuppression

## Abstract

**Context:**

Patients with end-stage renal disease can easily acquire a hepatitis C virus (HCV) infection via several ways. An HCV infection is difficult to treat after renal transplantation due to the conflicting actions of immunosuppressant therapy to maintain the function of the transplanted kidney and viricidal interferon (IFN) or ribavirin (RBV) treatment. Antiviral therapy requires great caution to avoid the complex and potentially fatal pharmacological effects. In this review, we examined clinical challenges and potential solutions for this specific scenario.

**Evidence Acquisitions:**

We searched Pubmed (NLM), LISTA (EBSCO), Web of Science (TS). The management of patients on waiting list, the indications and regimens about treatment were studied.

**Results:**

More than forty papers about this topic were found, including seven small clinical trials. International consensus has been reached to test patients awaiting renal transplantation. HCV detection after renal transplantation warrants careful consideration of when to initiate antiviral therapy. Treatment will begin immediately if deteriorating liver function increases the risk for loss of renal function. The choice of regimen depends on the patient's renal function and is individualized under close observation. The immunosuppressive regimen will be adjusted accordingly before antiviral therapy is initiated.

**Conclusions:**

The effects of modified antiviral therapy on these patients varies because of individual characteristics and disease state, and also because of the difficulty associated with conducting a large clinical trial to obtain statistically sound conclusions. The management before transplantation is important and when antiviral therapy needs to start, careful consideration of risks and benefits is needed before initiating this type of treatment.

## 1. Context

Patients with end-stage renal disease (ESRD) are required to undergo planned hemodialysis before renal transplantation is considered [1]. Dialysis is also necessary to improve a patient's quality of life while awaiting renal transplantation. Protracted exposure to externalities during hemodialysis increases the risk of infection and blood-borne disease, especially hepatitis C virus (HCV). The hypoimmunity characterizing these patients is an important predisposing factor for HCV infection [2]. If these patients become infected with HCV at the dialysis stage, clinicians face a big challenge managing the liver disease due to the conflicting pharmacological effects resulting from concomitant antiviral therapy and immunosuppressive therapy, which is absolutely required after renal transplantation.

Transplanted kidneys usually maintain their function with continuous administration of immunosuppressants. Commonly used immunosuppressive regimens include the tacrolimus (FK506) regimen (FK506 + mycophenolate mofetil [MMF] + prednisone) and the cyclosporine A (CsA) regimen (CsA + MMF + prednisone) [3]. These regimens often lead to massive viral replication, thereby accelerating the process of liver fibrosis and decreasing the efficacy of interferon (IFN) treatment [4][5]. High HCV load is also linked to other complications such as thyroid dysfunction, diabetes, essential mixed cryoglobulinemia (EMC), and idiopathic thrombocytopenia. HCV-associated glomerulonephritis is an example of a direct impact to transplanted kidneys caused by viremia. In addition to the kidneys, other organs such as the liver are also severely affected by these combined complications of HCV infection [6]. In several long-term clinical follow-up trials, patient mortality following renal transplantation varied from 8% to 28% due to liver failure; the incidence rate of mortality is 3 times greater in HCV infected organ recipients [7][8]. A follow-up on patient survival rates with and without HCV infection is shown in Figure. Patients positive for HCV demonstrate a lower survival rate [9][10]. In theory, antiviral therapy should be used to treat HCV; however, the immunomodulatory IFN component in antiviral treatments increase donor-specific alloantibodies and cause humoral rejection of the graft [11]. IFN also leads to additional side effects on hematopoietic cells. Therefore, the use of antiviral therapy in these HCV patients requires great caution to avoid the potentially fatal and complex pharmacological effects on the transplanted kidneys or other organs [6].

**figure:**
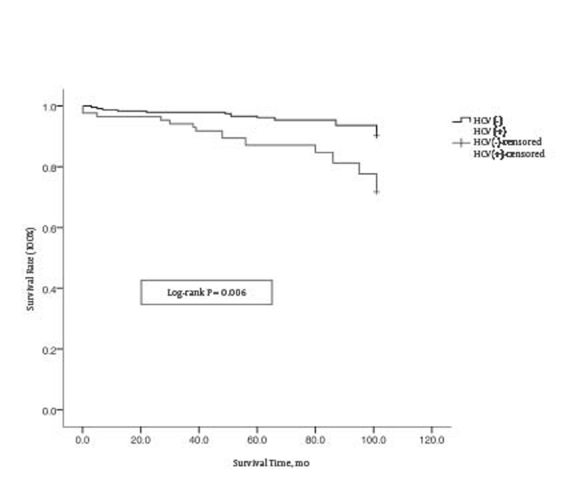
Patients Survival of Renal Transplantation Recipients by HCV Status

## 2. Evidence Acquisition

Pubmed (NLM), LISTA (EBSCO), Web of Science (TS) were searched with key words "Renal Transplantation", "Hepatitis C Virus", "Antiviral Agents", and "Immunosuppression" in recent 10 years, mostly recent 5 years. We also tried to obtain full articles and abstracts on the reference lists from retrieved documents. As the information about this topic was rare, small clinical trials and case reports were also included. The complexity of antiviral therapy, the management of patients on waiting list, the indications and regimens about treatment were studied.

## 3. Results

We found more than 40 papers including 7 small clinical trials about this topic. From the information we drew out following aspects.

### 3. 1. Evaluation and Management of Patients on the Renal Transplantation Waiting List

The treatment of HCV infection demands immune enhancement while kidney anti-rejection therapy requires immune suppression. An international consensus has been reached to conduct HCV testing on patients awaiting renal transplantation [12]. A similar system in Iran stratifies patients for eligibility: all candidates are tested for an HCV marker and alanine aminotransferase (ALT), and only the candidates whose results for both are normal qualify for the waiting list. Others are tested for HCV RNA; a patient with negative results qualifies, while a patient with positive results requires liver biopsy. If the liver biopsy indicates chronic hepatitis, antiviral therapy will be administrated until the patient shows sustained virological response (SVR), at which point the patient can be added to the list. If diagnosed with compensated cirrhosis, the patient will be considered for kidney and liver co-transplantation, a procedure for which it is extremely difficult to identify a donor. A liver biopsy indicating decompensated cirrhosis contraindicates transplantation [13][14]. The decision flow is shown in Diagram, in which the result of the liver biopsy result is the key factor in deciding whether a patient qualifies for transplantation. As a gold standard to determine liver conditions, a liver biopsy can detect or exclude complications such as occult cirrhosis. Moreover, it can guide the treatment plan for patients infected with HCV genotypes 1 or 4, which require a higher dosage and a longer course [15].One third of ESRD patients with HCV infection can achieve SVR via IFN administration, and thus, antiviral therapy is recommended for those patients. The combination of IFN with ribavirin (RBV) has further improved the rate of SVR. After treatment, patients who have attained SVR usually exhibit a long-term and durable virology, biochemistry, and histology response [16]. Several small clinical trials on drug administration in ESRD patients were analyzed by Perico et al. [17]. When 3 million units (MU) of common IFN was used weekly and 200 mg RBV was used 3 times per week for more than 24 weeks, more than 50% of patients achieved SVR-and the rate improved with lengthened treatment periods. The use of pegylated IFN further improved the SVR rate. The addition of RBV to the regimen may result in improved SVR, but with a greater risk of anemia [18][19][20]. More details of these clinical trial findings are shown in table 1.Although effective screening and treatment strategies have been implemented to help avoid dysfunction of the transplanted kidney, a big challenge in recrudescence and reinfection with HCV exists due to the side effects of IFN and RBV, which lead to the inability for most ESDR patients to accomplish the entire standard therapy regimen identified as "888." Patients also risk HCV infection from protracted blood exposure to externalities during renal transplantation surgery.

**diagram:**
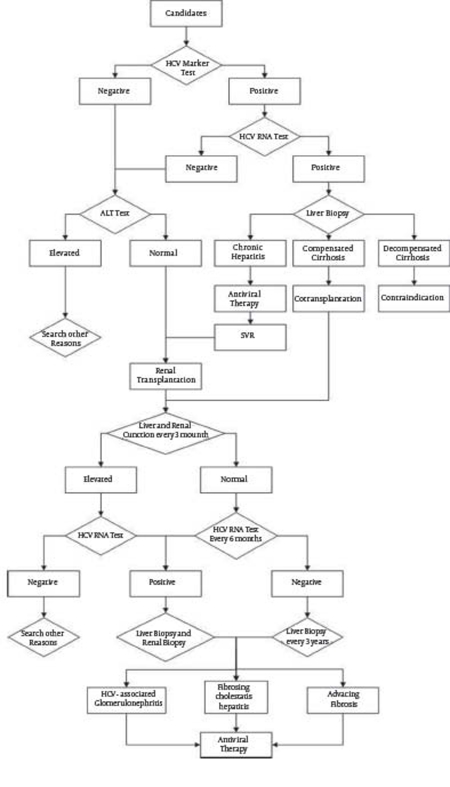
Flow Diagram for Patient Work-Up

One third of ESRD patients with HCV infection can achieve SVR via IFN administration, and thus, antiviral therapy is recommended for those patients. The combination of IFN with ribavirin (RBV) has further improved the rate of SVR. After treatment, patients who have attained SVR usually exhibit a long-term and durable virology, biochemistry, and histology response [16]. Several small clinical trials on drug administration in ESRD patients were analyzed by Perico et al. [17]. When 3 million units (MU) of common IFN was used weekly and 200 mg RBV was used 3 times per week for more than 24 weeks, more than 50% of patients achieved SVR-and the rate improved with lengthened treatment periods. The use of pegylated IFN further improved the SVR rate. The addition of RBV to the regimen may result in improved SVR, but with a greater risk of anemia [18][19][20]. More details of these clinical trial findings are shown inTable 1.

**table 1:** Drug Administration in End-stage Renal Disease (ESRD) Patients

	**Regimen**	**Dosage**	**Patients, No.**	**Duration, w**	**SVR **a** Rate, %**	**Drop-Out Rate, %**
Mousa DH et al. (2004) [18]	IFN a + RBV a	IFN, 3 MU/w; RBV, 200 mg, 3 times/ w	20	24 (n = 9) 48 (n = 11)	66 (24 w) 55 (48 w)	0
Rendina M et al. (2007) [19]	PEG-IFN α-2a + RBV	PEG-IFN α-2a, 135μg/ w; RBV, 200 mg/d	35	24 (genotypes non-1) 48 (genotype 1)	97	14 (1 patient was anemic)
van Leusen et al. (2008) [20]	PEG-IFN a α-2a + RBV	PEG-IFN α-2a, 135 μg/ w; RBV, 200 mg every other day	7	24 (genotypes 2 and 3) 48 (other genotypes)	71	0

^a^ Abbreviations: IFN, interferon; PEG-IFN, pegylated interferon; RBV, ribavirin; SVR, sustained virological response

Although effective screening and treatment strategies have been implemented to help avoid dysfunction of the transplanted kidney, a big challenge in recrudescence and reinfection with HCV exists due to the side effects of IFN and RBV, which lead to the inability for most ESDR patients to accomplish the entire standard therapy regimen identified as "888." Patients also risk HCV infection from protracted blood exposure to externalities during renal transplantation surgery.

### 3.2. Indication that Treatment Is Necessary After Renal Transplantation

The questions of whether an antiviral treatment can be initiated and how to implement remain controversial. In 2009, the guidelines regarding the diagnosis and treatment of hepatitis C, which were jointly amended by the American Association for the Study of Liver Diseases (AASLD), Infectious Diseases Society of America (IDSA), and American Society of Gastroenterology (ASG), declared that antiviral therapy based on IFN is not recommended for use in patients after renal transplantation due to the risk of acute rejection. This statement applies to patients with chronic kidney disease or to patients following organ transplantation. However, for patients with fibrosing cholestatic hepatitis, IFN is necessary for slowing liver cirrhosis and extending long-term survival [21][22][23]. Prior to this recommendation, Kidney Disease Improving Global Outcome (KDIGO) published a guideline in 2008 that suggests the use of antiviral therapy after renal transplantation if patients have advanced liver disease such as liver fibrosis; antiviral therapy is important to prevent liver-related death. The 2008 guideline also mentioned the necessity of this therapy for patients with fibrosing cholestatic hepatitis [12][21]. Moreover, some believe that treatment recipients with HCV-related glomerulonephritis should receive antiviral therapy actively; otherwise, the treatment will lead to deteriorating renal function or loss of the allograft [24].

To summarize these studies and guidelines, several major points regarding when to initiate antiviral therapy in renal transplant recipients infected with HCV are listed below:

1) Renal transplantation recipients with stable renal and liver function require strict observation rather than antiviral therapy. Liver and renal function tests should be conducted every 3 months and the viral load should be evaluated every 6 months. In particular, a liver biopsy should be repeated every 3 years [25].

2) Patients exhibiting fibrosing cholestatic hepatitis, advanced liver fibrosis, or HCV-associated glomerulonephritis should initiate antiviral therapy because it has known effects on long-term survival rates of both patients and allografts. Sustained suppression of necroinflammation may result in regression of cirrhosis which, in return, leads to decreased morbidity.

3) If antiviral therapy is deemed necessary, clinicians should assess first the risks and benefits and then inform the patients. Treatment initiation can only occur with both patient agreement and determination that the benefits outweigh the risks.

4) Because liver and renal function, the state of immunosuppression, and the maintenance regimen differ from patient to patient, as do the HCV complications and the impact of antiviral drug side effects, treatments should be individualized. All of these components require close evaluation before final decisions regarding antiviral treatment can be made.

In addition, the risk for acute rejection is higher during the first year after transplantation surgery [11]. Thus, it is strongly recommended to wait at least 1 year after the surgery to initiate antiviral therapy. Another study showed that antiviral treatment may yield a more effective response if stable renal function and no acute rejection occur during the first year after transplantation [26]. The management on this stage is also shown in Diagram.

### 3. 3. Treatment of Chronic HCV Infection

3.3.1. Standard Regimen

The 2009 edition of the diagnostic and treatment guidelines (AASLD/IDSA/ASG) for hepatitis C suggest that the standard treatment should be a combination of pegylated IFN α (PEG-IFN α) and RBV. The specific dosage and duration differ according to viral genotypes and patient response. Patients infected with HCV genotype 1 or 4 should expect a 48-week treatment duration consisting of 180 μg PEG-IFN α-2a per week, administered by hypodermic injection, and a daily weight-dependent dose of RBV, i.e. 1000 mg (body weight ≤ 75 kg) or 1200 mg (body weight > 75 kg). If PEG-IFN α-2b is used, a subcutaneous dose of 1.5 μg/kg should be administered weekly in addition to daily weight-dependent RBV, i.e. 800 mg (weight < 65 kg), 1000 mg (weight 65~85 kg), 1200 mg (weight 85~105 kg), or 1400 mg (weight > 105 kg). Patients infected with HCV genotype 2 or 3 should expect a basic course of 24 weeks with 800 mg RBV daily. If a patient cannot tolerate the side effects, 3 MU of IFN can be used 3 times per week. In addition, the dose of PEG-IFN α-2a can be reduced to 135 μg per week and PEG-IFN α-2b to 1μg/kg per week. Combined RBV is still acceptable when reduction and side effects such as anemia are closely monitored [22].

3.3.2. Reformed Regimen

What we had identified as stable renal function after transplantation was in fact not actually intact; non-impaired renal function includes either intact renal function or mild to moderate impairment due to HCV-related hemolysis, EMC, or other complications that are not in advanced stages [27][28]. Thus, the targets can be divided into 2 groups on the basis of renal function. Treatment with IFN alone is recommended for impaired renal function; it suppresses viral replication as well as imparts substantial therapeutic effects on cryoglobulin-associated mesangial proliferative glomerulonephritis. Thus, IFN alone can reduce kidney damage and improve the condition of microscopic hematuria and hypertension. The recommended dosage is IFN at 3 MU 3 times per week, PEG-IFN α-2a at 180 μg per week, or PEG-IFN α-2b at 1 μg/kg per week [17]. Since RBV mainly plays an immunosuppressive role in vivo, it was alone tested as a treatment to determine if it has any antiviral therapeutic value for reducing acute rejection. However, the risks associated with RBV alone far exceed any potential benefits for treating impaired renal function. RBV is processed mainly by the kidneys. Consequently, RBV, alone or in combination with another therapy, is not recommended for patients with impaired renal function [5].

Combined treatment is applicable for patients with complete renal function. According to actual patient glomerular filtration rates (GFR), RBV is used to supplement the recommended IFN dose. The ultimate goal is to achieve a blood concentration of 10-15 μmol/L over the long term. A trial was conducted previously that used a low-dose combination therapy consisting of the common 1 MU of IFN 3 times per week plus 600 mg of RBV per day [29]. The regimens described above and their extrapolated SVR rates are shown in Table 2[30][31][32][33]. In summary, the decision to initiate antiviral therapy depends on liver function; if liver function deterioration can potentially lead to loss of renal function, treatment should be initiated immediately. The renal function of each patient dictates the choice of regimen; therefore, the regimen should be individualized and patients should be closely monitored.

**Table 2:** Dose Adjustments Are Dependent on Renal Function

**Renal Function**	**Regimen**	**SVR a Rate Extrapolated from Small Trials, %**	**References**
Impaired			
29 mL/min /1.73 m ^2^ < GFR a < 60 mL/min/1.73 m ^2^	IFN, 3 MU a 3 times/ w	25	Therret E et al. (1994) [33]
	PEG-IFN^a^ α-2a, 180 μg/w	No trial yet	
	PEG-IFN α-2b, 1 μg/kg/w	No trial yet	
Complete			
GFR > 60 mL/min/1.73 m ^2^	IFN, 3 MU 3 times/wk + RBV^a^^b^	50	Fabrizi F et al. (2006) [30]
	PEG-IFN α-2a, 180 μg/wk + RBV	62	Montalbano M et al. (2007) [37]
	PEG-IFN α-2b, 1 μg/kg/wk + RBV	62	Schmitz V et al. (2007)[32]

^a^ Abbreviations: GFR, glomerular filtration rate; MU, million units; PEG-IFN, pegylated interferon; SVR, sustained virological response; RBV, ribavirin

^b^ Initial dose ranges from 200-800 mg daily according to the GFR to achieve long-term plasma concentrations of 10-15 μmol/L

### 3.4. Adjusted Immunosuppressive Regimen for HCV Patients After Renal Transplantation

Before initiating antiviral therapy, the immunosuppression regimen should be adjusted accordingly. When a patient is confirmed with HCV infection, it is reasonable to reduce the dosage of immunosuppressants to the lowest dose at which an immunosuppressive state can be maintained and liver damage is minimized. MMF metabolites can inhibit HCV replication effectively; thus, an improved long-term survival rate is achieved in patients without administering antiviral treatment. Further studies are needed to demonstrate how MMF metabolites are processed and removed when used alongside antiviral therapy. All current immunosuppressive regimens include MMF [34]. In animal experiments, CsA was able to inhibit HCV replication, independent of its immunosuppressive activity. This finding was corroborated by a retrospective cohort study in Germany, which demonstrated that the renal grafts in patients receiving CsA function better than in patients receiving FK506, but no significant differences were observed in viral replication and development of liver fibrosis [35]. Therefore, the CsA regimen may have better overall efficacy in HCV patients when initiated after renal transplantation [36]; however, an analysis on cost-effectiveness showed that the FK506 regimen is far more advantageous than the CsA regimen. Notably, this statement is currently a topic of considerable debate in the field due to the lack of prospective clinical studies.

## 4. Conclusions

Because of the specificity of patients and disease state, no consensus has been reached on the effect of modified antiviral therapy on renal transplantation patients, and large clinical trials to obtain statistically sound conclusions are difficult to conduct due to the unique nature of this disease. Nonetheless, several small trials have reported satisfactory curative effects, i.e. the SVR reached 62% with no harm to renal function [37]. Overall, the optimal regimen for HCV patients after renal transplantation remains out of reach. We need safer and more effective drugs or alternative therapies. The present advent of direct-acting antiviral (DAA) therapies are the biggest advance. These drugs specifically target the HCV-encoded nonstructural 3/4A (NS3/4A) serine protease and NS5B RNA-dependent RNA polymerase (RdRp) [38][39]. The serine protease NS3/4A is used by HCV for posttranslational processing and viral replication; it is the first successful target of DDAs. Protease inhibitors have been demonstrated to be potent inhibitors of viral replication. The first 2 DAAs are the linear serine protease inhibitors, telaprevir (TVR) and boceprevir (BOC) [40]. They both have finished phase III trials and will be available within the next year. However, resistance quickly develops when this type of compound is used alone [41]. Hence, the addition of this class of compound to the mainstream drug arena is a current developing paradigm.

Two phase II, multicenter studies of TVR combined with PEG-IFN α-2a and RBV in untreated patients infected with HCV genotype 1 resulted in the addition of TVR and significantly increased rates of SVR, albeit with enhanced discontinuation due to adverse events [42][43]. Rash and pruritus were the major adverse events in the groups that received TVR. They could be monitored during the treatment period. Both topical antiallergenic agents and topical and systemic antipruritic agents were used; however, severe rash necessitated corticosteroid treatment. Since immunosuppressive regimens already include prednisone, the rate of adverse events might be lower in patients after transplantation. In another randomized, stratified, partially placebo-controlled, partially double-blind, phase II study, we conducted a group study without RBV, i.e. TVR and PEG-IFN α-2a only for 24 weeks. However, a better SVR rate than the control group (which received the standard regimen) was achieved [44]. A regimen without RBV is possible; patients who cannot tolerate the side effects of RBV will benefit greatly from this regimen, especially the transplantation patients. BOC can also elevate the SVR rate in similar clinical trials [45], but anemia is a class effect of linear protease inhibitors, and it is more common with BOC compared to TVR [40]. Thus, the use of TVR might make women more suitable to frozen transplantation and both protease inhibitors call for a stable renal function and tolerance.

However, the actual effects of upcoming protease inhibitors will be examined in a special group of patients. Highly anticipated RNA interference therapies still remain in the research and development stage [46]. Given the high cost of current HCV regimens and numerous side effects, HCV-infected recipients have to face a double burden, i.e. financially and physically. Careful decision-making is needed before the initiation of such treatment and it often calls for a fine balance between risk and benefit.
